# Helps from flipped classroom in learning suturing skill: The medical students’ perspective

**DOI:** 10.1371/journal.pone.0204698

**Published:** 2018-10-02

**Authors:** Jen-Chieh Wu, Sheng-Chu Chi, Chien-Chih Wu, Yi-No Kang

**Affiliations:** 1 Department of Emergency Medicine, Taipei Medical University Hospital, Taipei, R.O.C. (Taiwan); 2 Department of Education, Taipei Medical University Hospital, Taipei, R.O.C. (Taiwan); 3 School of Medicine, College of Medicine, Taipei Medical University, Taipei, R.O.C. (Taiwan); 4 Department of Medical Education and Humanities, School of Medicine, College of Medicine, Taipei Medical University, Taipei, R.O.C. (Taiwan); 5 Department of Urology, Taipei Medical University Hospital, Taipei, R.O.C. (Taiwan); Universita degli Studi di Palermo, ITALY

## Abstract

**Background:**

Today, flipped classroom (FC) has been widely used in medical education. However, the effectiveness of FC remains controversial. The variation may cause by different subjects or different course design. Moreover, those studies did not explain how the association among different domains of learning objective was in FCs. The purpose of this study was to explore the help of learning domains from a FC of suturing skill in year-5 medical students.

**Design:**

This study determined sample size according to statistical power. A minimum number of 77 participants for regression analysis are needed. Therefore, this study enrolled 78 medical students in a 2-hour suturing course, which consisted of pre-class video and in-class instruction. Both simple and mattress suturing were taught. The students received an anonymous survey with questionnaire of Help from Instruction Questionnaire for Clinical Skills (HIQ-CS) after the course. The HIQ-CS was developed by medical education team according to Bloom's taxonomy, and its reliability was favorable (Cronbach's ^l = 0.839). Factor loadings among all items in the HIQ-CS was also favorable (0.790 to 0.849). This study determined consensus of students' perspective by median (Me) and interquartile range (IQR), and tested mediation among different learning domains by regression.

**Results:**

The results showed medical students agreed FC can help them in learning suturing (Me = 4, IQR = 1). The cognitive help (*β* = .526, *p* < .001) was completely mediated by psychomotor help (*β* = .399, *p* < .001) and affective help (*β* = .413, *p* < .001) to overall helps in FC. The affective help (*β* = .617, *p* < .001) was partially mediated by psychomotor help to overall helps in FC.

**Conclusions:**

FC may help students in learning suturing skill in different domains. Our model explains the cognitive help from FC provides an important foundation for the helps of other domains. Although the model should be examined by different curricula and measurements in future, the model of help from instruction in our study provided an innovated concept and framework in medical education.

## Introduction

The suturing is an essential skill for medical students. The Tomorrow’s Doctors that was published by General Medical Council indicated that medical students have to acquire suturing skill in undergraduate.[1] Teachers developed technology assisted curricula to help medical students to learn in recent years, especially flipped classroom (FC) [2–7]. Moreover, a good curriculum and assessment of suturing skill should involve multiple learning domains because an appropriate curriculum or assessment should cover multi-domains of learning objectives. For instance, the content of formal assessment in medical education, Objective Structured Clinical Examination, also includes the multi-domains of cognition, attitude, and skill [8]. Although the previous studies investigated how the innovate curriculum helps medical students to learn clinical skills among different learning domains, they were hard to portrayed the correlations or paths among learning domains. Most of the reports discussed the students’ performance, but few discussions regarded the factors on students’ performance.

Learning domains were structured by Benjamin Bloom and his colleagues very well after 1956, and the concept of learning domains were well-known as Bloom's taxonomy[9]. A revised version of the Bloom's taxonomy defined the cognitive domain, and made this model more practical and completed[10]. This taxonomy consists of cognitive domain, affection domain, and psychomotor domain, and it has been applied in education for a long time. Cognitive domain is related to mental skill, which involves knowledge and the development of intellectual skills[9]. Affection domain refers to the growth of feeling or emotional aspect, which includes how people deal with the thing emotionally[11]. Regarding to psychomotor domain, it mainly refers to the development of physical skills[12]. Similarly, the classification of educational goals involve knowledge goal, skill-based goal and affective goal[9]. Today, many models are similar to Bloom's taxonomy and have been widely used in nutrition, pharmacy and medical fields[13–15]. In addition, the association among knowledge, attitude, and practice are discussed in several studies. Moreover, those learning domains may affect each other. [16, 17]. This framework is applied not only in assessment, but also in curriculum development.

One of popular teaching and learning models in this age, FC, was developed since 1990s. This teaching and learning model is implemented in the medical education recently[18]. The FC consists of two forms of learning, pre-class and in-class[18]. In pre-class learning, instructors have to provide some targeted readings and video tutorials before the students take the course. In in-class learning, instructors should design learning activities including analyzing case studies and team-based learning exercise. This innovative model is an effective solution to improve student-centered learning[19], and the model may also benefit for students in learning medicine and healthcare[2–7]. Although some studies reported no score difference between FC and traditional course[20, 21], other studies proved that FC can significantly improve both non-knowledge and knowledge ability regarding surgery, cardiovascular, respiratory, and renal physiology[3, 6, 18, 19]. This heterogeneity among these studies might be caused by different subjects or course designs. Moreover, these studies did not explain how the correlation among different domains of learning objectives from FC was. Therefore, the effectiveness of FC is still controversial. In the meantime, the impact of FC on medical education still needs further research and discussion. The purpose of this study was to explore the correlations and paths among helps of learning domains in FC for suturing skill.

## Material and methods

**Fig 1:**
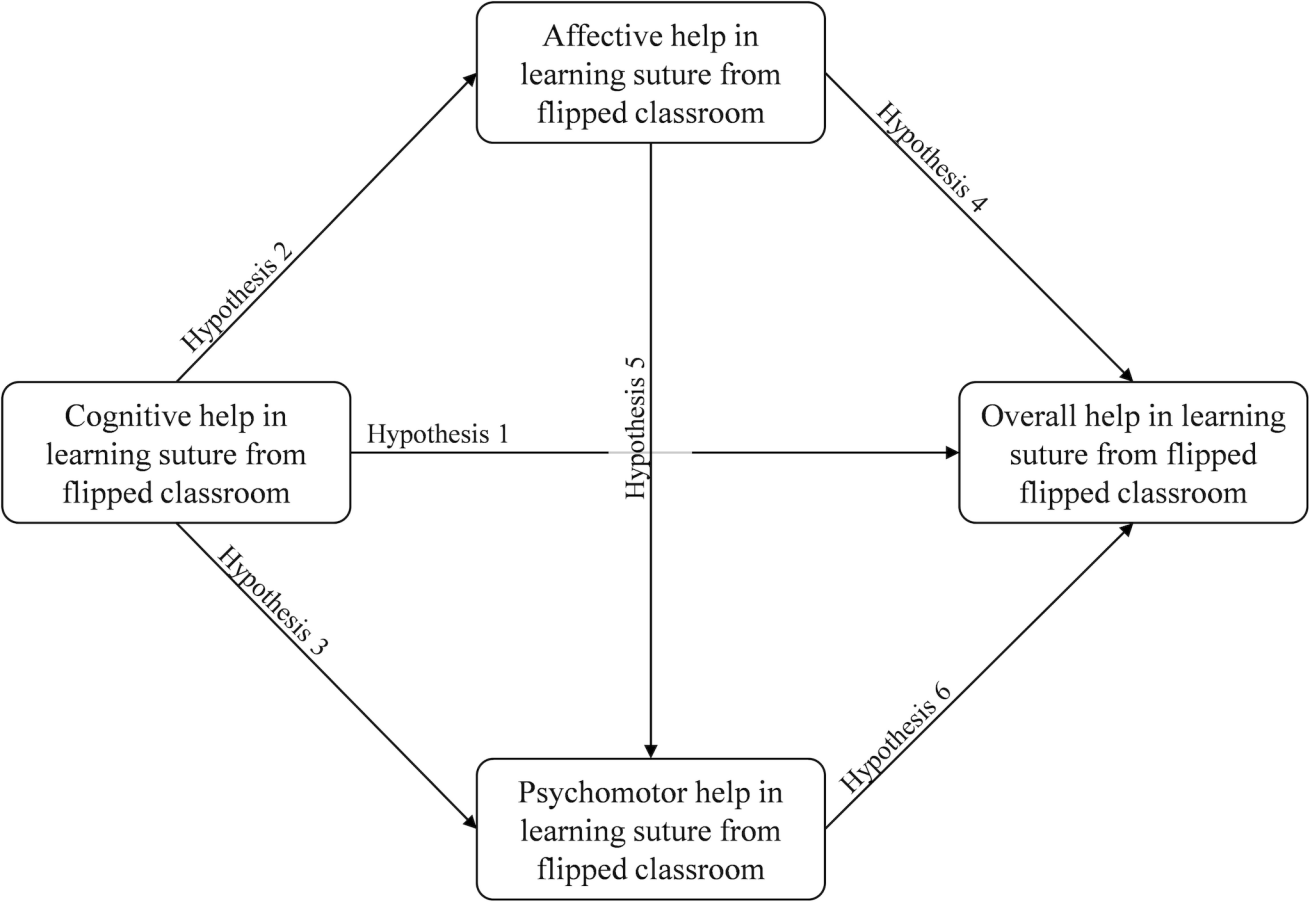
The research conceptual model depicting mediation of helps from flipped classroom.

We designed a FC for students to learn suturing skill and developed a questionnaire to ask them how the FC helps them in three learning domains (cognitive, affection, and psychomotor domains). Path analysis was conducted for exploring the association of three domains of learning in FC, and it was structured according to literature mentioned above. We assumed that FC can help medical students to learn suturing skill in the three domains. Moreover, the overall help from FC is determined by the helps of three domains. Therefore, this study structured a conception model of relationship among the awareness of “help from FC” in three learning domains and overall helps (Fig 1). The theoretical conception model that was drew from relevant studies included “cognitive help from FC”, “affection help from FC”, “psychomotor help from FC”, and “Overall helps from FC”. The relationship and hypotheses among the concepts were (i) “cognitive help from FC” can positively predict “overall helps from FC”; (ii) “cognitive help from FC” can positively predict “affection help from FC”; (iii) “cognitive help from FC” can positively predict “psychomotor help from FC”; (iv) “affection help from FC” can positively predict “overall helps from FC”; (v) “affection help from FC” can positively predict “psychomotor help from FC”; (vi) “psychomotor help from FC” can positively predict “overall helps from FC”. This study protocol had reviewed by TMU-Joint Institutional Review Board (S1 File).

### Participants

This study determined the sample size according to the rule of statistical power[22]. The determination of sample size was conducted by using G*Power 3.1 for Windows. For the study purpose, the significance level was set at 0.05, statistical power was set at 0.80, and *f*^2^ at 0.15 for medication analysis with linear regression. A minimum number of 77 participants for regression analysis are needed (S1 and S2 Figs). Seventy eight medical students participated in simulated course of clinical skills and filled the questionnaire of Help from Instruction Questionnaire for Clinical Skills (HIQ-CS) after the course. This number fulfilled the requirement.

### Procedure

We provided video tutorial (http://219.87.146.141/edu/htdocs/modules/tad_player/play.php?psn=4) for pre-class learning in the FC for suturing skill. Students who participated in the FC of suturing skill were asked to watch the tutorial video before the class. The pre-class video involved introduction to preparation of suture including materials and tools, explanation of suture procedure, and demonstration. The in-class part consisted of the hands-on practice, real-time feedback, and further demonstration by teacher. After the in-class activities, all medical students received an anonymous survey with Help from Instruction Questionnaire for Clinical Skills (HIQ-CS).

### Measures

The HIQ-CS was developed by medical education team according to taxonomy of educational objectives. The questionnaire was designed as a Likert 5-point scale, which ranged from 1 (strongly disagree) to 5 (strongly agree). Reliability and exploratory factor analysis were conducted to assess quality of the HIQ-CS. Cronbach’s α is a measure of internal consistency of a test[23]. The internal consistency reliability was favorable (Cronbach’s α = 0.839). The α value is higher than the threshold of 0.70 guided by scholars[24]. The results of exploratory factor and reliability analysis are shown in S1 Table.

Many social science studies use a cut-off for factor loadings of 0.30 to 0.40 (minimally acceptable), but values greater than 0.50 are generally considered necessary practical significance[24]. Communality in factor analysis should be greater than 0.50[24]. We used a cut-off of 0.50 for both factor loadings and communalities. The range of factor loadings among all the items are from 0.790 to 0.849, and all the items pasted the threshold. Therefore, we retained 4 items from the questionnaire because their scores meet the acceptable thresholds with value 0.50. Finally, these 4 items explained 67.69% of the variance in help of instruction from flipped classroom. We identified the variables from the 4 items according to Bloom’s taxonomy. For instance, we used statement “Flipped classroom helped me to understand the skill procedure very much.” to reflect the variable of “cognitive help from FC”. This connection is based on the definition of cognitive domain in Bloom’s taxonomy. To help students understanding a procedure can reflect “cognitive help”, because the understanding is related to knowledge and the development of intellectual skills. Similarly, we used the statement “Flipped classroom inspired me to do the skill with good attitude very much.” to reflect “affection help from FC”, and used the statement “Flipped classroom strengthened me to do the skill very much.” to reflect “psychomotor help from FC”.

### Data analysis

To investigate helps in learning suturing skill from FC, the present study followed consensus method that descriptive statistics should be used for determination of a special issue[25]. The threshold for agreement of questionnaire was set as 4 or higher on the 5-point scale in this study. The consensus criterion was interquartile range (IQR) 1 or less according to relevant literature[25].

Stepwise regression is conducted in a series of steps. In the series of steps, the predictors are selected automatic step by step based on its correlation with the dependent variable. The advantage of this approach is that it can be used to sort out the relevant explanatory variables from a set of candidate predictors[26]. Thus, the stepwise multiple regression method was employed to build a series of regression models for predicting the overall helps from FC across the three domains (predictors) of “learning helps” in learning suturing skill. To check for multicollinearity among predictors, tolerance and variance inflation factor (VIF), were computed. The cutoff for tolerance in this study is 0.10, and for VIF is 10[24].

We tested the hypotheses following the rules of mediation analyses. The basic and general causal steps approach for mediation analyses is based on relevant formula[27]. The formula for mediation analyses should follow the three steps: (i) Y = τX + e_1_, (ii) X_M_ = αX + e_2_, and (iii) Y = τ’X + βX_M_ + e_3_. The first step examines a mediation analysis assumption. The assumption requires a significant regression from predictor to criterion variable. The second step examines another mediation analysis assumption that required a significant regression from predictor to mediator. The third step consummates the mediation analysis with a regression from predictor and mediator to the criterion.

We analyzed data by using Statistical Product and Service Solutions version 19 for windows, and set probability, *p*-value, 0.05 for significance level in all statistical tests. Effect size of regression were performed as R-square. Another effect size that was recommended by Cohen is f-square when multiple regression is conducted in a psychological study[28]. The f-square can be estimated from R-square through a formula: f-square = R-square / (1-R-square). According to Cohen’s work, small, medium, and large effect sizes (f-square) can be defined by using f-square (.02, .15, and .35 respectively) and R-square (< .13, .13 to .25, and > .25 respectively) in a psychological multiple regression analysis. We also conducted further analysis for observed power by using Free Statistics Calculators with relevant formula and rule[29].

## Result

### Descriptive statistics and correlation among variables

All the items in the HIQ-CS were scored by 78 medical students. The majority of the students who participating in this study agreed that they got helps in learning suturing skill from FC (Me = 4.000; Mo = 4.000). Furthermore, they also agreed that FC provided them cognitive (Me = 4.000; Mo = 4.000), affective (Me = 4.000; Mo = 4.000), and psychomotor (Me = 4.000; Mo = 4.000) helps in learning suturing skill. These agreements met the threshold of consensus, and their IQRs were between 0.250 to 1.000 (Table 1). The results represented that students’ response were consistent.

**Table 1:** Descriptive statistics.

Variable	Minimum	*Maximum*	*Me*	*Mo*	*Q1*	*Q3*	*IQR*
Cognitive	2	5	4.000	4.000	4.000	4.250	0.250
Psychomotor	2	5	4.000	4.000	4.000	5.000	1.000
Affective	2	5	4.000	4.000	4.000	5.000	1.000
Overall	3	5	4.000	4.000	4.000	5.000	1.000

Note. Me, median; Q1, interquartile 1; Q3, interquartile 3; M, mean; SE, standard error.

The correlation coefficients among helps in learning suturing skill from FC were moderate. Cognitive help in learning suturing skill from FC and affective help in learning suturing skill from FC showed the highest correlation coefficient (*r* = .652). Cognitive help in learning suturing skill from FC and psychomotor help in learning suturing skill from FC showed the lowest correlation coefficient (*r* = .497) than other correlation coefficients (Table 2).

**Table 2:** Correlations among helps in different domain of learning suture from FC.

Variable	Psychomotor	Affective	Overall
Cognitive	.497***	.652***	.526***
Psychomotor	—	.511***	.609***
Affective	—	—	.617***

Note.

*** *p* < .001, two-tailed.

### Tests of mediation

The first step of medication analysis included regressions from cognitive help (predictor) and affective help (indirect mediator) to overall helps in learning suturing skill from FC (criterion variable). The result showed that cognitive help in learning suturing skill from FC accounted for 27.6% of the variance in predicted overall helps in learning suturing skill from FC (*F*_(1, 78)_ = 29.012, *p* < .001). A positive estimate for the standardized regression coefficient was obtained (*β* = .526, *p* < .001) (Table 3, Model 1). The affective help in learning suturing skill from FC (indirect mediator) also performed positive regression coefficient (*β* = .617, *p* < .001) that accounting for 37.2% of the variance in predicted overall helps in learning suturing skill from FC (*F*_(1, 78)_ = 46.596, *p* < .001) (Table 3, Model 2). These results confirmed the first step of mediation test that predictor and indirect mediator can predict criterion variable.

**Table 3:** The first step of mediation test: Predictor to criteria variable.

Independent	Dependent	Prediction			Model test	
variable	variable	*β*	95% *CI*	*t*	*R*^2^	*F*
**Model 1**	Overall				.276***	29.012
Cognitive		.526***	.278-.604	5.386		
**Model 2**	Overall				.372***	46.596
Affective		.617***	.370-.675	6.826		

Note. N = 78. CI = confidence interval.

*** *p* < .001, two-tailed.

In regressions for the second step of medication analysis, cognitive help served independent variable to predict two mediators (affective help and psychomotor help), and affective help (indirect mediator) served independent variable in prediction of psychomotor help (direct mediator). Cognitive help in learning suturing skill from FC can significantly predict psychomotor help (*β* = .497; *t* = 4.997; *p* < .001; 95% *CI* = .286-.622) and affective help (*β* = .652; *t* = 7.497; *p* < .001; 95% *CI* = .474-.817) in learning suturing skill from FC (Table 4, Model 3 and 4). Affective help in learning suturing skill from FC can significantly predict psychomotor help in learning suturing skill from FC (*β* = .511; *t* = 5.180; *p* < .001; 95% *CI* = .284-.639) (Table 4, Model 5).

**Table 4:** The second step of mediation test: Predictors to mediators.

Independent	Dependent	Prediction			Model test	
variable	variable	*β*	95% *CI*	*t*	*R*^2^	*F*
**Model 3**	Psychomotor				.247***	24.969
Cognitive		.497***	.286-.622	4.997		
**Model 4**	Affective				.425***	56.200
Cognitive		.652***	.474-.817	7.497		
**Model 5**	Psychomotor				.261***	26.835
Affective		.511***	.284-.639	5.180		

Note. N = 78. CI = confidence interval.

*** *p* < .001, two-tailed.

**Fig 2:**
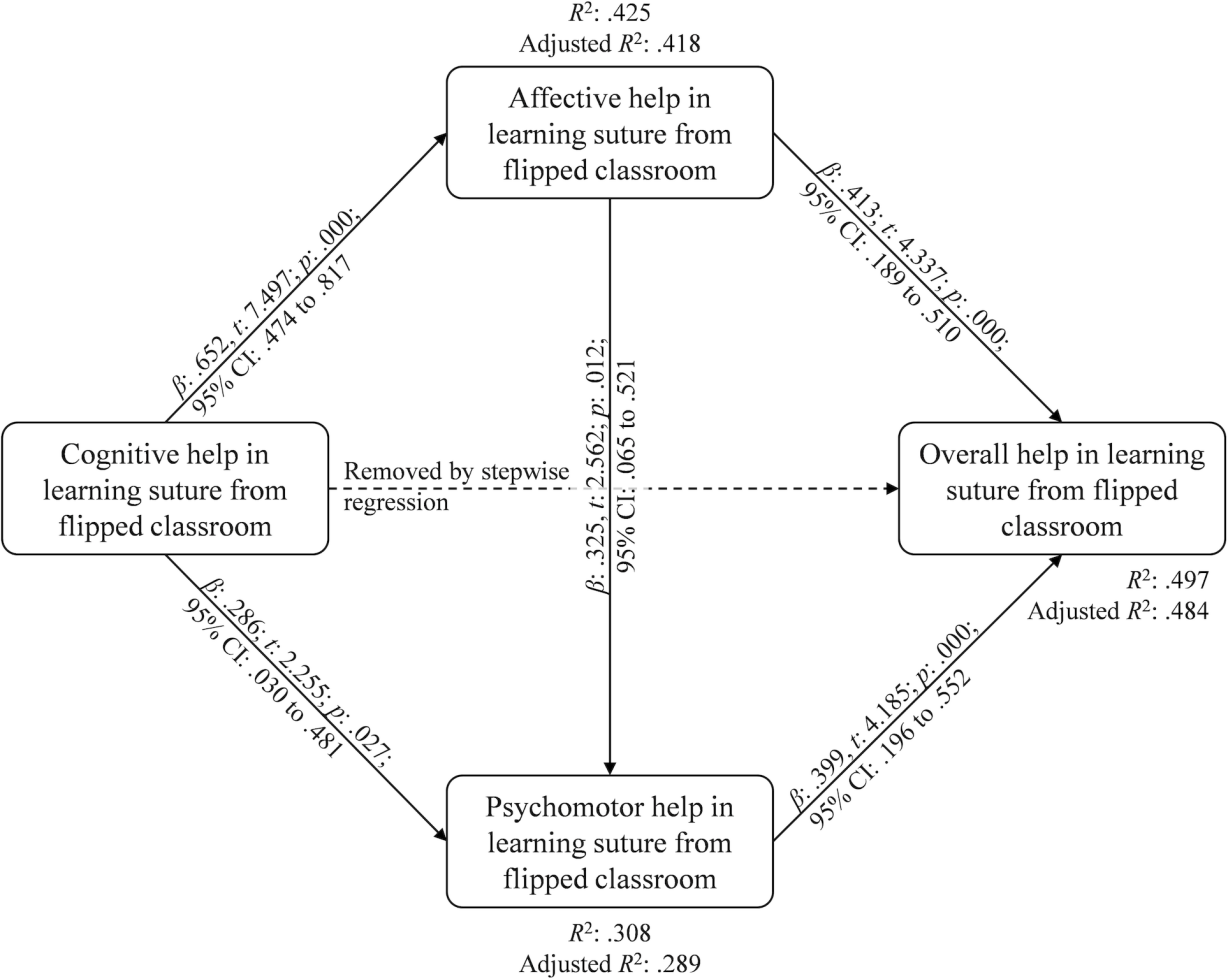
Path of mediation of helps from flipped classroom.

Results of regressions for the third step of medication analysis showed that cognitive help in learning suturing skill from FC was excluded from regression equation of overall helps by stepwise multiple-regression when two mediators were added in the model. Affective help (*β* = .417; *t* = 4.337; *p* < .001; 95% *CI* = .189-.510) and psychomotor help (*β* = .399; *t* = 4.185; *p* < .001; 95% *CI* = .192-.552) in learning suturing skill from FC can positively predict overall helps in learning suturing skill from FC with acceptable collinearity (VIF = 1.353), and they accounted for 49.7% of the variance in predicted overall helps in learning suturing skill from FC (*F*_(1, 78)_ = 37.118, *p* < .001) (Table 5, Model 6). In addition, results on stepwise multiple-regression of psychomotor help (direct mediator) showed that positive estimates for the standardized regression coefficient were observed from affective help (*β* = .325; *t* = 2.562; *p* = .012; 95% *CI* = .065-.512) and cognitive help (*β* = .286; *t* = 2.255; *p* = .027; 95% *CI* = .030-.481) in learning suturing skill from FC with acceptable collinearity (VIF = 1.739), and they accounted for 30.8% of the variance in predicted overall helps in learning suturing skill from FC (*F*_(1, 78)_ = 16.680, *p* < .001) (Table 5, Model 7). The summary of mediation analysis was performed in Fig 2.

**Table 5:** The third step of mediation test: Predictors and mediators to criterion variable and direct mediator.

		Prediction			*Model test*		Collinearity test	
Independent	Dependent							Condition
variable	variable	*β*	95% *CI*	*t*	*R*^2^*/*Δ*R*^2^	*F/*Δ*F*	VIF	index
**Model 6**	Overall				.497^a^***	37.118^c^		16.039
Cognitive		—	—	—	—	—	—	
Affective		.417***	.189-.510	4.337	.380^b^***	46.596^d^	1.353	
Psychomotor		.399***	.192-.552	4.185	.117^b^***	17.515^d^	1.353	
**Model 7**	Psychomotor				.308^a^***	16.680^c^		16.955
Affective		.325*	.065-.512	2.562	.261^b^***	26.835^d^	1.739	
Cognitive		.286*	.030-.481	2.255	.047^b^*	5.083d	1.739	

Note. N = 78. CI = confidence interval.

* *p* < .05, two-tailed

*** *p* < .001, two-tailed.

a. *R*^2^

b. Δ*R*^2^

c. *F*

d. Δ*F*.

## Discussion

This study figured out how helps from FC in learning suturing skill were noted by medical students, and found the associations among cognitive help, affective help, psychomotor help, and overall helps from FC. The cognitive help in learning suturing skill was completely mediated by psychomotor help and affective help to overall helps in FC. The affective help in learning suturing skill was partially mediated by psychomotor help to overall helps in FC. All the predictors performed positive effects on overall helps in FC. In overall, to a psychological study, the effect sizes in this mediation analysis were moderate to high except the effect size in “psychomotor help from FC” on “cognitive help from FC” in a combined regression model. Interestingly, the R-square in the regressions of “psychomotor help from FC” on “cognitive help from FC” was decreased when “cognitive help from FC” in the combined model. Moreover, this decreased R-square showed a very low effect size (.047) from a moderate effect size (.247). That is to say, the most of psychomotor help prediction from cognitive help is through affection help. According to these results, we can say that the FC increased medical students’ suturing skill not only by helping them to understand the procedure, but also by inspiring them to do procedure with good attitude. Furthermore, FC can inspire the medical students to suture with good attitude through helping them to understand suturing procedure.

Two mainly aspects of discussion regarding learning clinical skills were performance and students’ psychological process. Some of reports focused on the learning outcome including academic performance, or fair evaluation of clinical skills[3, 20, 21, 30, 31]. They discussed the various curricula for learning surgical skills including suturing. A study on laparoscopic suturing in FC evaluated numbers of knots and suturing quality by using modified checklist of the Objective Assessment of Technical Skills, and the study indicated that the FC group had higher pass rates in the knot-tying phase[3]. Another one study investigated the detrimental effects on learning laparoscopic learning skills, and the study aimed to explore the possibility to avoid the detrimental effects by giving the learning goal for the novice. Unfortunately, no significant difference between groups were observed, and the performances in the both groups were similar to each other[31]. A prospective trial implemented FC to surgical clerkship, and the differences between pre-test and post-test were found across all curricula. In addition, the study also suggested that the FC might increase students’ interest in surgical career[20]. Although these studies provided summative learning outcomes, they were hard to provide information regarding to how was the learning process. Main function of summative assessment is to assess the effectiveness and efficiency of a learning program[32]. Those studies can proof how good their teaching programs are, but they were hard to really realize which part of instruction should be modified according to summative learning outcomes.

Furthermore, these studies were conducted for outcomes regarding academic score or performance, but seldom discussed how curricula helped students in their learning process from students’ perspective. For educational progress, it is important to take students’ perspective into consideration in curriculum evaluation[33, 34]. The present study tried to fulfill this need by performing how FC helped students in their learning process, especially in psychological processes.

Researchers in educational psychology believe that mature learner are active and constructive, and they also believe that learner can monitor, control, and regulate their own cognition, motivation, behavior, and features of their learning. For instance, in self-regulated learning (SRL) theory, students can set learning goal to strive for in their learning process[35, 36]. The concept of SRL raised recently in medical education, and the reports on SRL applied to medical education are growing[37]. Moreover, the SRL theory also indicated that a mature learner can effectively use various strategies to reach their learning goals. One of those strategies, resource management strategy, is help-seeking, which means mature students can distinguish what actually help them in their learning[38, 39]. Medical students as university students are mature learners, and they may know which learning form can benefit themselves. Therefore, the results in the present study that were based on medical students’ perspective were reasonable, and the results provided whole picture of the associations among cognitive help, affective help, psychomotor help, and overall helps from FC in learning suturing skill.

The other studies focused on students’ psychological process during or after participating curricula or learning activities regarding to knowledge and skills[40, 41]. One common psychological process in those studies is self-efficacy. One study explored the impact on clinical skills and self-efficacy after the curriculum, and it found improvements on self-efficacy and clinical skills[40]. Another study evaluated self-efficacy of medical student after a team-based learning for end-of-life care, and the study showed increases in both learning confidence and critical thinking skills[41]. The present study also focused on students’ psychological process, and we structured psychological process according to relevant educational theory. This kind of results can provide analytical outcomes to evaluate learning process, and provide information regarding to how teaching program should be modified.

Video tutorial, one of the pre-class activities of FC was valuable from students’ perspective, and it helped students learning in many FCs[14]. The in-class part of FC including real-time advices and demonstration from teacher were also important in helping students to learn skills[3]. The pre-class video tutorial may help students especially in cognitive domain, and the in-class instruction may enhance students’ affective and psychomotor domain. Similarly, our study designed pre-class video to establish students’ basic knowledge of suturing skill, and the present study designed in-class instruction to enhance their affective and psychomotor domains of suturing skills through hands-on practice, real-time feedback, and further demonstration. Then, the medical students agreed that FC can help them in learning knowledge of suturing skill. The cognitive (knowledge) help can predict affective and psychomotor helps from FC though it cannot predict overall helps from FC directly. The overall helps from FC was predicted by affective and psychomotor domains of suturing skill directly. The result of our mediation analysis portrays a phenomenon, and it implies the importance of real-time feedback and further demonstration from teacher in the in-class part of FC. That is to say, the pre-class video met students’ knowledge (cognitive domain) learning need, and it cannot replace in-class instruction and activities from teacher. This phenomenon may be due to the complexity of suturing steps, because the instructional video is hard to demonstrate them thoroughly.

### Limitation

There are some limitations in our study. First, we designed the mediation model according to Bloom’s taxonomy. The Bloom’s taxonomy covers various learning domains, and those learning domains might be weighted according to purpose in different curricula. The present study sampled from a suturing course, and the psychomotor domain (skill-based) may be weighted more than the other two domains. Therefore, our results can provide the insight for educators who would like to design skill-based curriculum. It is not sure whether the outcome of knowledge-based curriculum can find the same results. Second, the present study completed in a single institution with relatively small sample size. The result cannot completely symbolize all medical students, and its generalizability is limited to other medical students, especially those in other countries.

## Conclusion

The FC may help students learning suturing skill in different domains, and its helps are associated with each other. Our model explains that the cognitive help from FC provides an important foundation for the helps of other domains in learning suturing skill according to medical students’ perspective. The correlations among helps from instruction, self-efficacy of skills, self-efficacy of learning skills, and real performance should be evaluated in further study. Although the model should be examined by different curricula and measurements in future, the model of help from instruction in the present study provided an innovated concept and framework in medical education.

## Supporting information

S1 Table Results of descriptive and reliability analysis for the questionnaires and items. (DOCX)

S1 Fig X-Y plot for sample size estimation in G*Power. (TIF)

S2 Fig Central and noncentral distributions for sample size estimation. (TIF)

S1 File IRB.pdf. (PDF)
