# Supplementary material for: Helps from flipped classroom in learning suturing skill: The medical students’ perspective
Source: PLoS One. 2018 Oct 2;13(10):e0204698. doi: 10.1371/journal.pone.0204698 (PMC6168146; doi:10.1371/journal.pone.0204698)
Supplement: S1 Table — (DOCX) [file pone.0204698.s001.docx]

## S1 Table

| Results of descriptive and reliability analysis for the questionnaires and items | | | | | |
| --- | --- | --- | --- | --- | --- |
|  | | Factor analysis | | Cronbach’s α | |
| Items | | Communality | Factor loading | If item delete | Overall |
| HIQ-CS | |  |  |  | 0.839 |
| 1. | Flipped classroom helped me knowing skill procedure a lot. | .660 | .812 | .801 |  |
| 2. | Flipped classroom strengthened me doing the skill very much. | .624 | .790 | .815 |  |
| 3. | Flipped classroom promoted me to do the skill with good attitude a lot. | .721 | .849 | .776 |  |
| 4. | Over all, flipped approach improved my skill capability. | .703 | .838 | .789 |  |
