# Supplementary figures and images for: Helps from flipped classroom in learning suturing skill: The medical students’ perspective
Source: PLoS One. 2018 Oct 2;13(10):e0204698. doi: 10.1371/journal.pone.0204698 (PMC6168146; doi:10.1371/journal.pone.0204698)

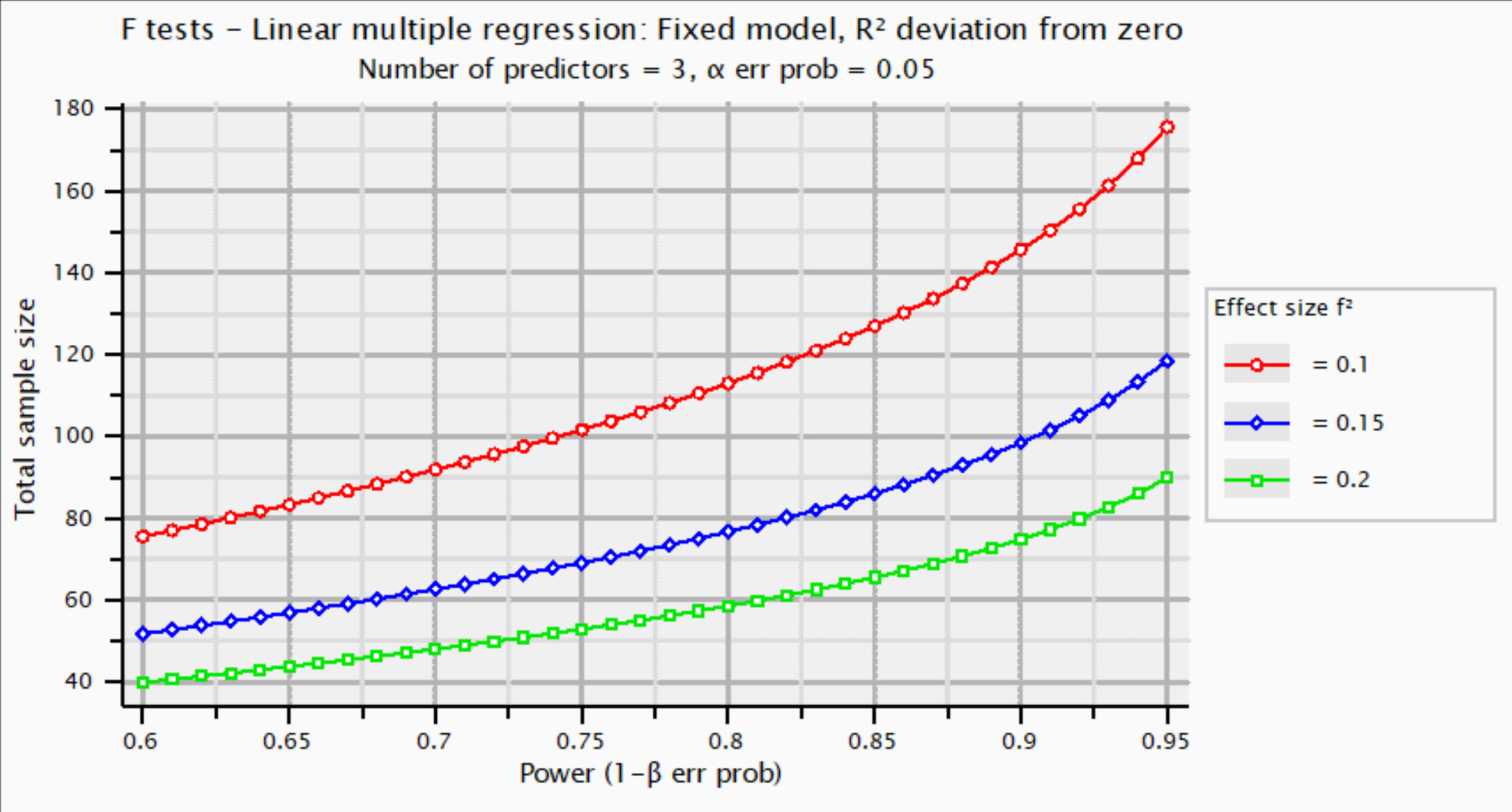

Supplement: S1 Fig — (TIF) [file pone.0204698.s002.tif]

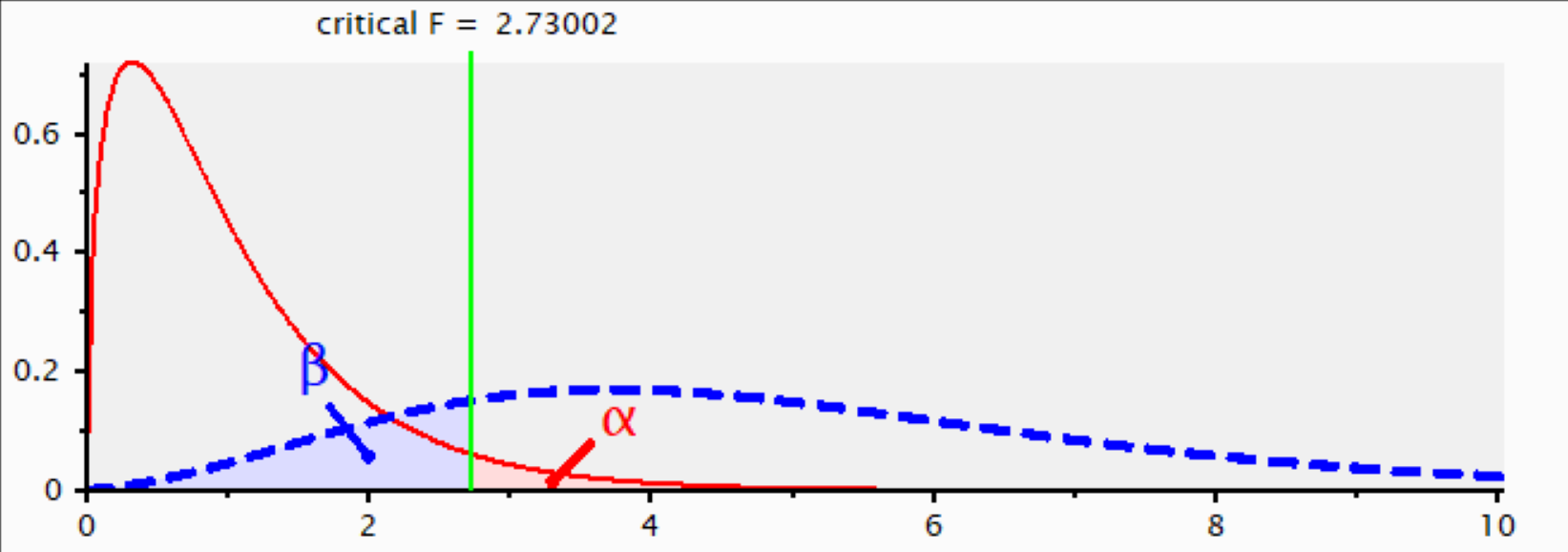

Supplement: S2 Fig — (TIF) [file pone.0204698.s003.tif]
