# Supplementary material for: Helps from flipped classroom in learning suturing skill: The medical students’ perspective
Source: PLoS One. 2018 Oct 2;13(10):e0204698. doi: 10.1371/journal.pone.0204698 (PMC6168146; doi:10.1371/journal.pone.0204698)
Supplement: S1 File — (PDF) [file pone.0204698.s004.pdf]

臺北醫學大學暨附屬醫院聯合人體研究倫理委員會

TMU-Joint Institutional Review Board

研究計畫免予審查證明

開立日期：民國106年11月30日

計畫編號：N201711071
計畫名稱：翻轉教學應用於擬真臨床技能學習之成果初探
計畫主持人：吳建志
研究機構：臺北醫學大學附設醫院

上述計畫經本會確認得免予審查，將於**第106-12-4次**會期(會議日期：106年12月21日)核備，特此證明。

主任委員：

Certificate of TMU-JIRB

Issue Date: 2017/11/30

TMU-JIRB No.: N201711071
Protocol Title: A pilot analysis of simulated clinical skills learning in flipped classroom
Principal Investigator: Wu, Chien-Chih
Study Site:Taipei Medical University Hospital

The above study has been confirmed Exemption by TMU-Joint Institutional Review Board in meeting #106-12-4 (date:2017/12/21).

Chairman:

Kuan-Jen Bai

臺北醫學大學暨附屬醫院
聯合人體研究倫理委員會
Taipei Medical University
Joint Institutional Review Board

本會組織與執行皆符合適用法規
The TMU-Joint Institutional Review Board performs its functions according to written operating procedures and complies with GCP and with the applicable regulatory requirements.
